# Early and delayed assessments of quantitative gait measures to improve the tap test as a predictor of shunt effectiveness in idiopathic normal pressure hydrocephalus

**DOI:** 10.1186/s12987-016-0044-z

**Published:** 2016-11-22

**Authors:** Masatsune Ishikawa, Shigeki Yamada, Kazuo Yamamoto

**Affiliations:** 1 Rakuwa Villa Ilios, 186 Jyrakumawari-nishimachi, Nakagyouku, Kyoto, 604-8402 Japan; 2 Normal Pressure Hydrocephalus Center, Rakuwakai Otowa Hospital, 2 Chinjicho, Yamashinaku, Otowa, Kyoto, 607-8062 Japan; 3 Department of Neurosurgery, Rakuwakai Otowa Hospital, 2 Chinjicho, Yamashinaku, Otowa, Kyoto, 607-8062 Japan

**Keywords:** Hydrocephalus, Aged population, Tap test, Gait disturbance

## Abstract

**Background:**

To improve the diagnostic performance of the cerebrospinal fluid (CSF) tap test (TT), early and delayed assessments of gait were performed after the removal of 30 ml of CSF in patients with probable idiopathic normal pressure hydrocephalus. Assessments of gait included the 3-m timed up and go test (TUG), and the 10-m walk in time (10Ti) and in step (10St) tests.

**Methods:**

Quantitative data for the TUG, the 10Ti, and the 10St were obtained before CSF removal and on days 1 and 4 after removal of 30 ml CSF. CSF shunt surgery was performed in 61 patients within one month after the TT. The gait outcome was assessed 3 months after surgery. The area under the curve (AUC), sensitivity, specificity, and cutoff values were computed for the TUG, the 10Ti, and the 10St on day 1 and day 4 using receiver operating characteristic (ROC) curve analysis.

**Results:**

The positive response rate in three measures on day 4 was equal to or higher than the values on day 1. Times were reduced significantly in the TUG and the 10mTi tests between baseline and both days 1 and 4 after TT. No significant differences were noted in the number of steps for the 10St test. The percent change in TUG on day 1 had the highest AUC value among all other variables (0.808). Although this was not statistically different from other variables in the TUG and the 10Ti, it had a good balance of high sensitivity (78.3%) and high specificity (80.0%), with a cutoff value of 11.3%. The change in the measured value in the day 1 TUG had the second highest AUC value at 0.770. The variables on day 4 tended to have high specificities of around 90%, although their sensitivities were low.

**Conclusions:**

The percent change of TUG on day 1 showed the highest diagnostic accuracy. Delayed assessments on day 4 were not superior to those on day 1. Thus, the TUG on day 1 is useful as a simple quantitative measure for predicting shunt effectiveness.

## Background

Idiopathic normal pressure hydrocephalus (iNPH) is a disorder resulting in abnormal gait, cognition, and urination in the aged population [1, 2]. CSF shunt surgery is effective in improving the symptoms of iNPH, especially those concerning gait [2]. The cerebrospinal fluid (CSF) tap test (TT), which involves the removal of 30–50 ml of CSF, is useful for the diagnosis of iNPH [3]. However, its diagnostic accuracy has been reported to vary between low and high [4–8]. This may be due to inconsistencies in various factors, such as the volume of CSF removed, the timing of the assessment, qualitative vs. quantitative assessments, and the use of single vs. multiple examiners. Virhammar et al. [9] recommend early assessment within 24 h of CSF removal. However, delayed improvement of symptoms is often observed. Recently, Schniepp et al. [10] reported a maximal increase in gait velocity 24–48 h after the TT using quantitative measures of gait. Assessment of gait is performed using clinical grading scales in most studies. However, categorical scales lack reliability. Since quantitative measures for gait are more reliable and have good objectivity, we investigated the clinical usefulness of quantitative measures of gait. We used the timed up and go test (TUG), the 10-m walk in time (10Ti), and the 10-m walk in step (10St) tests. In order to improve the diagnostic performance of the CSF TT, we focused on the following clinical questions: (1) When is a better time for the assessment: day 1 or day 4? (2) Which is the best measure of gait among the above three popular measures? (3) Which is the best variable to use for the measured values: the change in the measured value, or its percent change? Diagnostic performances of the three measures were investigated using multiple receiver operating characteristic (ROC) curve analyses.

## Methods

### Study population

This study was approved by the institutional board in Rakuwakai Otowa Hospital, Kyoto, Japan (Rakuoto1023). To study the usefulness of the TT, it was performed in 101 patients with possible iNPH as defined by the Japanese guidelines for iNPH [11] from January 2012 to December 2015 in Rakuwakai Otowa hospital. Brain and spine magnetic resonance imaging (MRI) was performed in all patients. There was a positive response to TT in 75 of the patients. A positive response was defined as an improvement of one point or more on the Japanese iNPH grading scale (GS) [12] or an improvement of 10% or more above baseline in quantitative measures of the TT made according to a proposal of the Japanese guidelines for iNPH [11]. Among the 75 patients with a positive response, 61 patients with subsequent shunt surgery were included in this analysis. Patients who did not receive surgery due to patient’s unwillingness and 5 patients who had severe gait disturbance requiring support at assessment, were excluded. Among the 61 patients, 51 had both early and delayed assessments of the TT. The remaining ten patients did not undergo the delayed assessment of the TT, due to the patient’s unwillingness or difficulty with the examiners’ time schedules. However, to exclude the possibility of selection bias, statistical analyses were performed on all 61 patients who had undergone surgery and in whom quantitative measures of gait could be obtained without any support.

### MRI

The brain MRI studies included T2-weighted images, T2-star weighted images, fluid attenuation inversion recovery images, constructive interference in steady state (CISS) images, and magnetic resonance angiography. MRIs with T2-weighted images were carried out for both the lumbar and the cervical spine. Characteristic findings of iNPH on MRI are known to include disproportionately enlarged subarachnoid space hydrocephalus (DESH) [2]. DESH consists of ventriculomegaly, tight high convexity, and enlarged subarachnoid space. Each of these components was categorized into marked, fair, or none. Patients classified as DESH were marked for all three components. Patients classified as incomplete DESH had one fair and two marked categories among the three components. Patients with other classifications were considered non-DESH [13]. CISS findings from this study are reported elsewhere [14].

### Tap test

For the TT, 30 ml of CSF were removed via a lumbar tap. Quantitative assessments of gait and cognition were performed before the lumbar tap, on day 1 (within 24 h of the tap), and on day 4. Fifty-seven patients were assessed within 24 h (18–24 h) to fit with the time schedules of rehabilitation staff and 4 patients were assessed within 3 h after the CSF removal.

Physiotherapists examined patients quantitatively using the TUG (s), the 10Ti (s), and the 10St (steps). On the TUG, the examination was performed in the rehabilitation room and physiotherapists measured time while a patient rises from an arm chair, walks 3 m, turns, walks back and sits down again, with maximum speed [15]. Assessments were done twice for each measure and the faster time or fewer steps were adopted. For the 10Ti and 10St, the patient was also requested to walk with maximal speed. The Mini-Mental State Examination and the Frontal Assessment Battery were carried out by speech therapists. Measured values, changes in measured values from baseline, and percent changes were compared between day 1 and day 4 groups for statistical significance (Table 1).

**Table 1:** Clinical summary of patients

Contents	Early assessment	Delayed assessment
Number of patients	61	51
Age^a^	76.9 ± 6.0	76.6 ± 5.5
Male preponderance^b^	73.8	70.6
Gait/cognition/urination^b^	100/80.3/73.8	100/82.3/76.4
Onset of gait disturbance <1 year/improved at 1 year	32/24	26/20
MRI of DESH/incomplete DESH/non-DESH^b^	55.7/23.0/21.3	60.8/17.6/21.6
Gait improvement on tap test^b^	91.8	96.1
Number of VP shunt/LP shunt	44/17	39/12
Improvement of gait at 3 months^b^	73.8	76.5%

Patients with early assessment on day 1 after CSF removal and delayed assessment on day 4 were subjected in study. Patients whom support was necessary for assessment of gait were not included in this study. The clinical characteristics were comparable among the two groups

*DESH* disproportionately enlarged subarachnoid-space by MRI, *VP* ventriculoperitoneal, *LP* lumboperitoneal

^a^ Years: mean ± standard deviation

^b^ Percentages

### Shunt surgery

Shunt surgery, consisting of either a ventriculoperitoneal (VP) shunt (n = 44) [2] or a lumboperitoneal (LP) shunt (n = 17) [16], was performed in all patients within one month after the TT. Selection of the type of shunt was based on the presence or absence of severe lumbar or cervical canal stenosis, the patient’s preference, and the surgeon’s preference. The Codman Hakim programmable valve with Siphonguard or Medtronic Strata NSC system, were used. The outcome was assessed 3 months after the shunt surgery using the gait domain of the Japanese iNPH GS by the senior author (MI) at the outpatient clinic. The outcome at 3 months was chosen to largely reflect the results of surgery, whereas if delayed for 6 months or 1 year the outcome could have been confounded by further modifications of comorbidity and nursing care. Improvement of gait at three months was defined as a one-point-or-larger improvement on the GS [11].

### Statistical analyses

For the analysis of the means of continuous data, we used the Wilcoxon signed-rank test to perform nonparametric statistics. Comparisons of percentages of categorical data were performed using the Chi square test. Comparisons among the three groups (Baseline, Day1 and Day4) were performed using repeat measures analysis of variance (ANOVA) with Bonferroni post hoc comparison. Diagnostic accuracy of each variable was calculated using area under the curve (AUC), sensitivity, specificity, and cutoff levels obtained using receiver operating characteristic (ROC) curves. The significance level was set at 0.05. All statistical analyses were performed using the open-source software R (version 3.0.1; R Foundation for Statistical Computing, Vienna, Austria; http://www.R-project.org). ROC analyses were performed using the package program pROC (version 1.8) [17].

## Results

Clinical background data are summarized in Table 1. Early assessment on day 1 after TT was performed in 61 patients with a mean age of 76.9 years. Delayed assessment on day 4 was performed in 51 patients with a mean age was 76.6 years. Gender, symptoms, MRI findings, and positive response on the TT were comparable between the groups with or without delayed assessment and no statistical differences were noted.

**Fig. 1:**
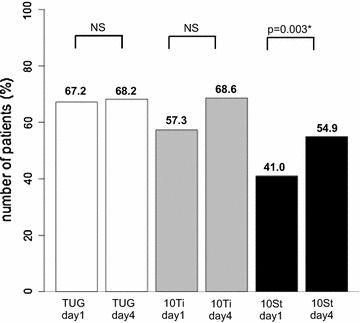
Number of patients showing a positive response to the three tests on days 1 and 4 after the tap test. The percentage of positive responses was comparable between day 1 and day 4 for the timed up and go (TUG) and the 10-m walk in time (10Ti) tests but significantly higher for the 10-m walk in step (10St) test at day 4. The number of patients was 61 on day 1 and 51 on day 4

**Fig. 2:**
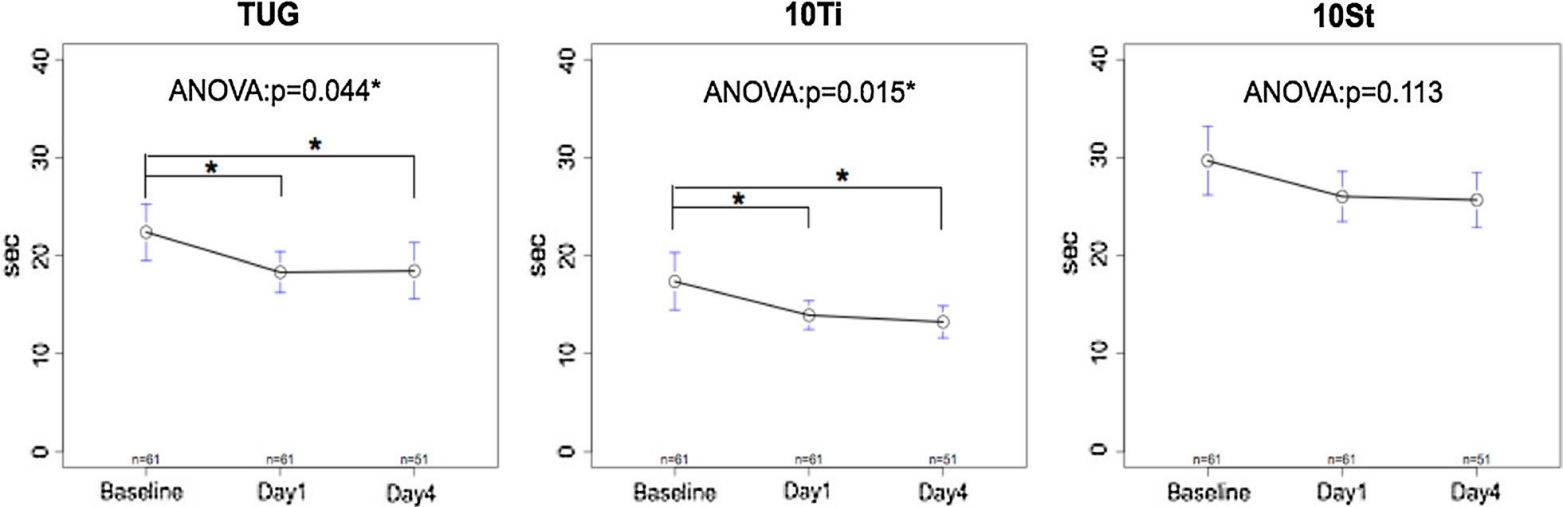
Graphs showing the results for the three tests at baseline (before the tap test), and on day 1 and day 4 after the tap test. All measures decreased in value from baseline on day 1 and day 4. ANOVA revealed statistical significance for the TUG and the 10Ti, but not for the 10St. Post-hoc analysis indicated a significant decrease in time between the baseline and day 1, and between the baseline and day 4 in the TUG and the 10Ti tests, but not between day 1 and day 4. Values are mean ± confidence level

Positive response rates in gait which was defined as improvements of 10% or more over baseline in the TUG, the 10Ti, and the 10St on day 1 and day 4 are depicted in Fig. 1. Positive response rates on day 4 were equal to or higher in the three measures compared to day 1, with statistical significance in the 10St test. In the TUG and the 10Ti tests, repeat measures ANOVA analysis of the measured values at baseline and on days 1 and 4 revealed a statistically significant difference (Fig. 2). Post hoc analysis revealed significantly reduced times between baseline and day 1 and between baseline and day 4 in the TUG and 10Ti tests (Fig. 2). No statistical differences were noted in the 10St test.

**Fig. 3:**
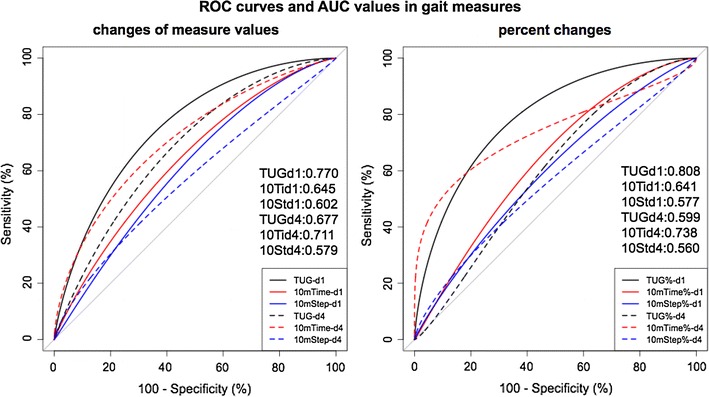
Receiver operating characteristic (ROC) curves and area under the curve (AUC) values on day 1 (*solid lines*) and day 4 (*dotted lines*). ROC curves for changes of measured values from baseline are left and ROC curves for percent changes are on the right. The AUC for TUG on day 1 was highest for both ROC curves

The ROC curves of the three measures on days 1 and 4 are depicted for changes in the measured values (Fig. 3, left) and their percent changes (Fig. 3, right). AUC values were computed using the ROC analyses. The measured change and the percent change of TUG on day 1 both had higher AUCs. The AUC, sensitivity, specificity, and cutoff values are tabulated in Table 2. Each measure has a different sensitivity and specificity and a different AUC.

**Table 2:** Receiver operating curve analysis

Examination day	Measures (change in time or step number from baseline)	AUC	p	Sensitivity (%)	Specificity (%)	Cutoff
Day 1 (N = 63)	TUG (s)	0.770	0.249	76.6	75.0	2.0
	10Ti (s)	0.645	0.102	63.8	68.8	0.8
	10St (step)	0.602	0.027*	36.1	93.8	4.5
Day 4 (N = 52)	TUG (s)	0.677	0.283	43.9	90.9	5.0
	10Ti (s)	0.711	0.383	63.4	81.2	2.3
	10St (step)	0.579	0.053	34.1	90.0	5.5

Examination day	Measures (change in percentage from baseline)	AUC	p	Sensitivity (%)	Specificity (%)	Cutoff (%)
Day 1 (N = 63)	TUG (%)	0.808	–	78.3	80.0	11.3
	10Ti (%)	0.641	0.193	63.0	66.6	9.4
	10St (%)	0.577	0.016*	41.3	86.7	13.6
Day 4 (N = 52)	TUG (%)	0.599	0.109	73.2	50.0	10.0
	10Ti (%)	0.738	0.483	63.4	90.0	15.8
	10St (%)	0.560	0.036*	41.5	80.0	17.0

ROC curve analysis showed the AUCs, sensitivities, specificities and cutoff values. The percent change from baseline of AUC on day 1 was regarded as a control and compared with other variables. Note the TUG on day 1, both for change in time and change in percentage, showed the highest AUC

Statistical differences were noted in most of 10Sts (*), but not in other variables of the TUG and the 10Ti

**Fig. 4:**
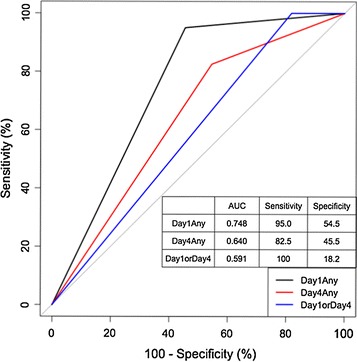
Positive response in any of three assessment measures. Positive response in any of three assessment measures on day1 (*black line*), day 4 (*red line*) or either day 1 or day 4 (*blue line*) showed high sensitivity but their specificities were low

Among these measures, the percent change in the TUG on day 1 had the highest AUC value (0.808) and a sensitivity of 78.8% and a specificity of 80.0%. The cut-off value for the TUG was 11.3%. Although this AUC value was significantly different from most variables of 10St, no statistical differences were noted between the variables of the TUG and the 10Ti. The second highest AUC (0.770) was noted for the change in the measured value of the TUG on day 1. The TUG on day 1 had a sensitivity of 76.6%, a specificity of 75.0%, and a cutoff value of 2.0 s. Overall, the sensitivity of the TT ranged from 34.1 to 78.3%, while its specificity ranged from 50.0 to 93.8% (Table 2). Thus, the TT tended to have a relatively low sensitivity and a high specificity. The variables on day 4 tended to have high specificities of around 90%, although their sensitivities were low. Among these variables, the 10Ti had fairly high AUCs of above 0.7. When a positive response was defined as positive in any one of the three assessment measures, the sensitivities became very high but specificities became low and their AUCs did not exceed the AUC of the TUG on day 1 (Fig. 4).

## Discussion

Improvement of gait is the most frequent and useful finding after surgical treatment of iNPH. TT is a popular method to predict and assess shunt effectiveness and is not very invasive. Regarding the diagnostic accuracy of the TT, the specificity has been reported to be high with low sensitivity [4–6], although high sensitivity was also reported [7]. In our previous cooperative study of iNPH, named SINPHONI, the gait domain had a high specificity of 80% and a relatively low sensitivity of 51.3% [8]. We have often observed delayed improvement in gait after CSF removal. This observation led us to study the clinical significance of delayed assessment for the prediction of shunt effectiveness. Recently, Schniepp et al. reported a delayed improvement over 3 days after CSF removal [10]. Thus, the timing of assessment after CSF removal is an important issue in the tap test. In this study, delayed assessment was performed on day 4. The positive response rate on day 4 was equal to or higher in three measures than on day 1. The TUG and the 10Ti had statistically significant reductions in time both on day 1 and on day 4. Multiple ROC curve analyses indicated that the percent change of the TUG on day 1 had the highest AUC value, 0.808, which is a fairly high value. The TUG had a good balance between sensitivity and specificity. The second highest AUC of 0.770 was noted in the change of the measured value in the TUG on day 1. Most of measures on day 4 had fairly high AUCs, but they did not exceed the percent change of the TUG on day 1. We also examined the possibility of a high AUC if any of the three measures was positive. Any measure that was positive on day 1 had a fairly high AUC value. However, none were superior to the percent change of the TUG on day 1. Thus, the TUG on day 1, either as a percent change or as a change in the measured value, was shown to be useful for predicting shunt effectiveness.

In this study, most of the assessments on day 4 had high specificities around 90%. High specificity indicates that the number of false positive cases is small, which is favorable from the standpoint of recommendation for surgery. Similarly, high sensitivity indicates that the number of false negative cases is small, which is favorable for selecting candidates from the general population. In this context, the day 4 study is still useful if a positive response is noted. This is because of the high specificity of the test on day 4.

The second issue to consider is the modality of the examination of gait. The 3-m TUG and the 10-m walk are popular choices. Stoltz et al. reported increased velocity due to an enlarged stride length after the removal of 30 ml of CSF [18]. In this study, gait was simply assessed using time and step number. A decrease in the time and an increased velocity was confirmed using the TUG and the 10Ti. However, the decrease in the number of steps on the 10St was not confirmed. Instrumented walkway assessments could provide more objective information. Williams et al. reported that velocity, double support time, and cadence improve significantly after CSF drainage in patients with iNPH in assessments using an instrumented walkway [19]. Schniepp et al. also reported an increase in walking speed in various tasks performed on the walkway [10]. They noted maximal improvements of dual task paradigms on days 2 and 3, and recommend that assessments of gait be performed on day 2 or day 3.

In this study, a positive response in quantitative measures was defined as 10% improvement from the baseline according to the proposal of Japanese iNPH guidelines. This proposal is based on clinical experience. In this study, the cut-off values in TUG, 10 m walk in time and in step on day 1 were close to 10%. Thus, present study reveals that the guidelines’ proposal is appropriate for gait.

Third issue that we investigated is the assessment of improvement. Change of gait after CSF removal is usually assessed using various kinds of qualitative grading scales, such as Kiefer’s scale [20] and the Japan iNPH grading scale [7]. The scores obtained using these scales may be different depending on the individual examiner. Assessment of the baseline state may vary, and assessment of changes after the interventions may be more variable. As a result, the reproducibility of these scales may be low. Furthermore, the qualitative scores obtained are difficult to compare among the different grading systems. We have observed differences in the scores as determined by physicians vs. physiotherapists, even when using the same grading scale [21]. The former tended to place more emphasis on changes in symptoms, while the latter emphasized changes in daily activities. Quantitative measures are more reliable and can be compared between reports. However, measured values may only represent a part of the patient’s disability.

This study has several limitations. This is a retrospective study in a single third-referral hospital. The outcome was assessed by a neurosurgeon using the qualitative scale of the Japan iNPH GS. The improvement rate of 76.9% may be relatively low. This is assessed using a subjective score and may cause some bias in ROC curve analyses. Scores on the iNPH GS after the TT may be different when assessed by different examiners. To overcome these problems, well-designed grading scales to assess the degree of severity and subsequent changes with high inter-rater agreement are necessary. The instrumental walkway may provide more information regarding gait changes. This study did not include cognitive data. Cognition may be associated with gait improvement, as reported by Allali et al. [22]. We tried the dual-task walk in some patients, but no consistent findings were noted. Further study is necessary to obtain a high prediction rate for shunt effectiveness.

## Conclusions

To improve the diagnostic performance of the CSF TT, early and delayed assessments in gait were performed after the removal of 30 ml of CSF from patients with suspected iNPH. Quantitative data from the TUG, the 10Ti, and the 10St were obtained before CSF removal and on day 1 and day 4 after CSF removal. The gait outcome was assessed 3 months after CSF shunt surgery. ROC curve analysis indicated that the highest AUC value (0.808) was observed for the percent change in the TUG on day 1. The TUG on day 1 showed the highest diagnostic accuracy with the sensitivity of 78.8% and the specificity of 80%, and the cutoff value was 11.3%. Delayed assessment on day 4 was not superior to that on day 1. Thus, the TUG on day 1 is useful as a simple quantitative measure for predicting shunt effectiveness.

## Abbreviations

ANOVA: analysis of variance
AUC: area under the curve
CISS: constructive interference in steady state
CSF: cerebrospinal fluid
DESH: disproportionately enlarged subarachnoid-space hydrocephalus
GS: grading scale
iNPH: idiopathic normal pressure hydrocephalus
LP: lumboperitoneal shunt
MRI: magnetic resonance imaging
ROC: receiver operating characteristic
TT: tap test
TUG: timed up and go test
VP: ventriculoperitoneal shunt
10Ti: 10-m walk in time
10St: 10-m walk in step
